# Supplementary material for: Paradoxical Changes: EMMPRIN Tissue and Plasma Levels in Marfan Syndrome-Related Thoracic Aortic Aneurysms
Source: J Clin Med. 2024 Mar 8;13(6):1548. doi: 10.3390/jcm13061548 (PMC10970932; doi:10.3390/jcm13061548)
Supplement: Supplementary file 1 [file jcm-13-01548-s001.zip › jcm-2872597-supplementary.pdf]

Supplemental file 1: Detailed Patient Demographics

For MFS TAA tissue, sex was distributed: 63.64% male and 36.36% female. One individual identified as Hispanic while all others were non-Hispanic. The average age was 31±2 years. Non-TAA control aortic tissues were harvested from the ascending aorta of heart transplant donors or recipients; mean ages are 58±6 years; aortic diameters are 3.8±0.2 cm with no prior diagnosis of MFS. For MFS TAA plasma, sex distribution was 54.79% male and 45.21% female. The average age was 38.48±16.98 years. Non-TAA control plasma was 50.82% male 49.18% female. The average age was 42.08±16.63 years. Further details are listed below:

Control Plasma Demographics Summary

- Sex:
    - Male (n = 62)
        - Age: 42.11 ± 15.72
    - Female (n = 60)
        - Age: 42.05 ± 17.66
- Ethnicity:
    - Unknown (n = 19)
        - Age: 43.37 ± 16.55
    - American Indian/Alaska Native (n = 6)
        - Age: 41.83 ± 10.87
    - Asian (n = 2)
        - Age: 19.5 ± 2.12
    - Black/African American (n = 3)
        - Age: 34.33 ± 14.57
    - Hispanic/Latino (n = 12)
        - Age: 33.92 ± 19.84
    - Other (n = 1)
        - Age: 61
    - White/Caucasian (n = 79)
        - Age: 43.66 ± 16.25

Marfan TAA Plasma Demographics Summary

- Sex:
    - Male (n = 40)
        - Age: 39.52 ± 17.72
    - Female (n = 33)
        - Age: 37.22 ± 16.21
- Ethnicity:
    - Asian (n = 1)
        - Age: 66.86
    - Black/African American (n = 2)
        - Age: 46.73 ± 12.93
    - Native Hawaiian/Pacific Islander (n = 2)
        - Age: 28.18 ± 6.00
    - Not Specified (n = 32)
        - Age: 35.18 ± 16.31
    - White (n = 36)
        - Age: 40.74 ± 17.42

SUPPLEMENT - DETAILED METHODOLOGY

Multiplex Suspension Array (MSA): MSAs were used to measure MMPs and TIMPs in human plasma and aortic tissue (FCSTM07 and LKTM003, R&D Systems) following manufacturer's instructions. In short, aortic tissues were homogenized in 4°C buffer (volume 1:6 weight/volume) containing 10 mM cacodylic acid pH 5.0,0.15 M NaCl, 10 mM ZnCl2, 1.5 mM NaN3, and 0.01% Triton X-100. Homogenates were centrifuged (800 x g, 10 min, 4°C) and protein concentration was determined by BCA assay and 20 µg of total protein was analyzed. Plasma specimens were thawed on ice, centrifuged (16,000 x g, 4 min, 4°C) and diluted according to the manufacturer instructions. For MMP-2 and -9, samples were diluted 1:100; for all other MMPs, samples were diluted 1:10; for TIMPs, samples were diluted 1:20. The above assays were quantitated using a Bio-Rad Bioplex-200 analyzer. Results were compared to a standard curve from the same plate. Using analyte concentrations, fold changes from control values were calculated.

Immunoblotting analysis: Thawed tissues were transferred to 4°C homogenization buffer (buffer volume 1:6 weight/volume) containing 137 mM NaCl, 2.7 mM KCl, 8 mM Na_2HPO_4, and 2 mM KH_2PO_4, and disrupted in a 1mL glass tissue grinder. Homogenates were centrifuged (10,000 rpm, 2 min, 4°C) and the supernatants were used for immunoblotting. Ten µg, determined by BCA assay, of each aortic homogenate were loaded on a 4–15% gradient, bis-tris gel and fractionated by electrophoresis. Proteins were transferred to nitrocellulose membranes (0.45 µm, Bio-Rad, Hercules, CA) and incubated in antisera specific for MT1-MMP (1:2000; ab38971, Abcam) which was diluted in 5% bovine serum albumin (BSA)/PBS. A secondary peroxidase-conjugated antibody (primary antibody species-specific) was applied (1:5000, 5% BSA/PBS) and signals were detected with chemiluminescent substrate (SuperSignal West Pico PLUS Chemiluminescent Substrate, Thermo Fisher Scientific, Waltham, MA) and recorded using the Bio-Rad ChemiDoc XRS. Band intensity was quantified using ImageJ. Gels were normalized using two methods (further described in Results: *MT1-MMP protein abundance was increased in*

MFS TAA tissue). After performing MT1-MMP analysis, the nitrocellulose membranes were washed with a harsh stripping buffer containing 20 mL SDS 10%, 12.5 mL Tris HCl - pH 6.8 - 0.5 M, 67.5 mL distilled water, 0.8 mL ß-mercaptoethanol. The membrane was then incubated in antisera specific for GAPDH (1:2500; Cat#31460, Thermo Fisher Scientific, Waltham, MA) diluted in 5% bovine serum albumin (BSA)/PBS. The second method of normalization was total protein densitometry form 20-120 kDa, using ultraviolet light activated trihalo. This normalization method provided the least amount of variation and was therefore used for data analysis.

Cell Culture: Primary aortic fibroblast cell lines (n=3) were established from human aortic biopsies of non-syndromic aneurysmal thoracic aortas using an established outgrowth technique as previously described.[3] The isolated aortic fibroblasts were maintained in complete fibroblast-specific growth media (Fibroblast Growth Media 2; C-23020, PromoCell, Heidelburg, Germany) with added 10% fetal bovine serum (97068-085), and gentamicin (0.5 mg/mL; 15710-064, Gibco) at 37°C in 5% CO2. Aortic fibroblasts from culture passages 2 to 10 were used in the following studies.

In Vitro Transfection: Human aortic fibroblasts were transfected for 18 hours at 50% confluence in 75 cm^2 flasks (430641U; Corning) using jetPRIME transfection reagent (114-15, Polypolus-transfection) according to the DNA transfection protocol with either 10 µg MT1-MMP Over Expression vector (MT1-OE; OriGene, MC219253) or transfection reagent alone. Transfected fibroblasts were lifted with Tryp-LE (12604021, Thermo Fisher Scientific), and 200,000 cells per well were seeded in 6-well culture dishes. Serum-free culture media was exposed to the transfected cells and collected 48 hours later. Media was centrifuged at 2,000 x g for 10 minutes to remove debris and stored at -20°C.

Alteration of MT1-MMP Localization: MT1-MMP can be differentially located in aortic fibroblasts; protein kinase C-mediated phosphorylation of the C-terminal tail of MT1-MMP causes translocation of MT1-MMP from the membrane to endosomal vesicles in the cytoplasm.

Treatment with phorbol 12-myristate 13-acetate (PMA), which activates both classical and novel PKCs, results in enhanced internalization of MT1-MMP. Conversely, translocation of MT1-MMP can be inhibited with Röttlerin, a PKC-δ-specific inhibitor. PMA was used to induce internalization of MT1-MMP in isolated fibroblasts while Röttlerin was used to confine it to the cell surface. Primary human aortic fibroblasts were seeded in 6-well plates with 200,000 cells per well and cultured in complete growth media. Cells were cultured (37°C, 5% CO_2) until >75% confluence and treated for 24 hours: PMA (100 nM), Röttlerin (3 μM), or vehicle control (DMSO, 1:1000). After transfection, the cells were exposed to serum-free culture media. The conditioned serum-free media was collected after 24 hours exposure, centrifuged at 2,000 x g for 10 minutes to remove debris, and stored at -20°C.

Soluble EMMPRIN ELISA: A human EMMPRIN ELISA (ab219631, Abcam) was performed on the conditioned media according to protocol. In brief, 50 μL of standards or sample were added to the appropriate wells and exposed to 50 μL of antibody cocktail. After 1 hour of incubation at room temperature, the liquid was aspirated, and the wells were washed three times with 350 μL of the recommended wash buffer. Wells were incubated in 100 μL of the recommended development solution for 10 minutes; development was stopped with an equal volume of the recommended stop solution. The plate was measured at an OD value of 450 nm.
